# SARS CoV-2 Infection among Health Care Workers from Different Health Care Facilities in Western Norway: A Prospective, Cross-Sectional Study

**DOI:** 10.3390/v14122652

**Published:** 2022-11-28

**Authors:** Bård Reiakvam Kittang, Bjørn Blomberg, Marianne Sævik, Jan Stefan Olofsson, Bergen COVID-19 Research Group, Nina Langeland, Rebecca Jane Cox

**Affiliations:** 1 Department of Nursing Home Medicine, Municipality of Bergen, 5020 Bergen, Norway; 2 Department of Clinical Science, University of Bergen, 5007 Bergen, Norway; 3 Department of Medicine, Haraldsplass Deaconess Hospital, 5009 Bergen, Norway; 4 Department of Medicine, Haukeland University Hospital, 5021 Bergen, Norway; 5 Influenza Centre, Department of Clinical Science, University of Bergen, 5007 Bergen, Norway; 6 Department of Pediatrics, Haukeland University Hospital, 5021 Bergen, Norway; 7 Department of Research and Innovation, Haukeland University Hospital, 5021 Bergen, Norway; 8 Broegelmann Research Laboratory, Department of Clinical Science, University of Bergen, 5007 Bergen, Norway; 9 Department of Safety, Chemistry and Biomedical Laboratory Sciences, Western Norway University of Applied Sciences, 5063 Bergen, Norway; 10 Department of Microbiology, Haukeland University Hospital, 5021 Bergen, Norway

**Keywords:** COVID-19, SARS-CoV-2 antibodies, HCW, PPE

## Abstract

*Background:* Comparative data on COVID-19 among health care workers (HCWs) in different health care settings are scarce. This study investigated the rates of previous COVID-19 among HCWs in nursing homes, hospitals and a municipal emergency room (ER). *Methods:* We prospectively included 747 HCWs: 313 from nursing homes, 394 from hospitals and 40 from the ER. The diagnosis of COVID-19 was based on serological evidence of SARS-CoV-2 antibody positivity and self-reported RT-PCR positivity prior to inclusion. Information regarding age, sex and exposure to SARS-CoV-2 infection was collected. *Results:* A total of 4% (11/313) of nursing home HCWs and 6% (28/434) of HCWs in hospitals/the ER tested positive by serology and/or RT-PCR (*p* = 0.095). Fewer HCWs in nursing homes had occupational exposure to SARS-CoV-2 compared to those in hospitals/the ER (16% vs. 48%, *p* < 0, 001), but nursing homes had a higher proportion of HCWs with occupational exposure using partial/no PPE (56% vs. 19%, *p* < 0.001). Nevertheless, no significant differences in the risk for COVID-19 were found in relation to the rate of occupational exposure (*p* = 0.755) or use of inadequate PPE (*p* = 0.631). *Conclusions:* Despite a small sample size, the risk for COVID-19 among HCWs did not appear to be related to the type of health care facility, rates of occupational exposure or use of PPE.

## 1. Introduction

Occupational exposure to Severe Acute Respiratory Syndrome Coronavirus-2 (SARS-CoV-2) among HCWs in nursing homes and other primary care facilities, as well as in hospitals, has been substantial, particularly in areas with a high incidence of infection [1,2]. In the early phases of the pandemic, there was a lack of personal protective equipment (PPE) and a staffing shortage, especially in nursing homes [3,4,5]. This, along with the often-atypical clinical presentation of Coronavirus disease 19 (COVID-19) among nursing home residents [6] and difficulties in maintaining infection control measures among wandering and/or agitated persons with dementia, might put HCWs in nursing homes at a higher risk for occupational exposure to COVID-19 than those in hospitals. Most studies on the seroprevalence of SARS-CoV-2 among HCWs have been performed in hospitals, and comparative serological data on HCWs in primary care facilities and hospitals are relatively scarce [1,7,8,9]. A study from Iraq showed a significant association between the seroprevalence of SARS-CoV-2 and PPE training in primary care HCWs [10]. However, this potential relationship has to date primarily been investigated in hospitals and university-based health care systems [11,12,13].

The main aim of the present study was to explore the rates of COVID-19 among HCWs, in relation to type of health care facility, exposure to SARS-CoV-2 and the use of PPE.

## 2. Materials and Methods

### 2.1. Study Population and Clinical Data

**Figure 1:**
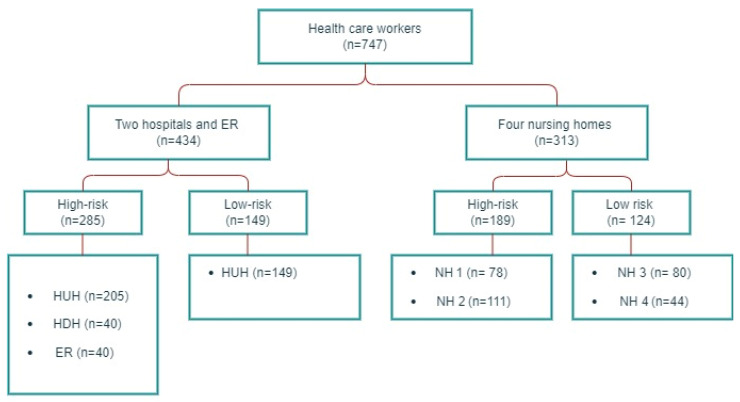
Study population flow chart. HUH: Haukeland University Hospital, HDH: Haraldsplass Deaconess Hospital, ER: Bergen Municipality Emergency Room, NH: nursing home.

In the period from 28 September 2020 to 11 January 2021, the study participants were recruited from four nursing homes, two hospitals and an emergency room (ER) in the city of Bergen, Norway. Nursing home HCWs (*n* = 313) were consecutively enrolled from three long-term institutions and one primary care facility with a mixed population of short-term and long-term residents. Two of the nursing homes had outbreaks of COVID-19 before inclusion, and were defined as high-risk institutions, and two institutions without prior outbreaks as low-risk facilities. HCWs from hospitals (*n* = 394) were consecutively enrolled from the tertiary care center Haukeland University Hospital and the local hospital Haraldsplass Deaconess Hospital, in the Bergen Health Region. High-risk departments treated patients with COVID-19, and low-risk departments were not involved in the clinical care of such patients. All participants from the ER (*n* = 40) were working in a SARS-CoV-2 testing facility. A flow chart of the study population is depicted in Figure 1. Preventive measures in wards treating patients with COVID-19 included the isolation of patients/residents with a suspected or confirmed SARS-CoV-2 infection and the use of PPE upon testing and patient/resident care during the infection course. Furthermore, HCWs with symptoms compatible with COVID-19, along with their close contacts, were tested and home-isolated awaiting test results.

All HCWs included in the study provided a peripheral venous blood sample for serology at the time of inclusion, and were invited to complete a questionnaire including age, sex, and non-occupational and occupational exposure to SARS-CoV-2. Participants with positive serology, regardless of any results of prior RT-PCR-testing, along with seronegative participants who gave anamnestic information of a previous positive RT-PCR result, were defined as having been infected with SARS-CoV-2.

### 2.2. Laboratory Methods

The diagnosis of COVID-19 was based on self-reported SARS-CoV-2 RT-PCR positivity on samples from nasopharyngeal swabs as part of the national testing program where HCWs were prioritized, and on serological evidence of SARS-CoV-2 antibody positivity. Serum samples were allocated a unique identification number and frozen before running in the ELISA. A two-step ELISA was used to confirm SARS-CoV-2-specific antibodies, firstly through screening for antibodies to the SARS-CoV-2 receptor-binding domain (RBD) antigens from the Wuhan-1 virus and then a confirmatory SARS-CoV-2 Wuhan-1 virus spike IgG ELISA as previously described [14].

### 2.3. Statistical Analysis

Dichotomized variables were presented as percentages, and differences were assessed by a χ-square test, and presented with odds ratios (ORs), 95% confidence intervals (CI) and *p*-values. For variables with a low number of observations (<5), a Fisher’s exact test was used. Statistical analysis was performed in R version 4.2.1 (The R Foundation for Statistical Computing, http://www.R-project.org, accessed on 4 September 2022, using RStudio version 2022.07.1 (Boston, MA, USA).

## 3. Results

**Figure 2:**
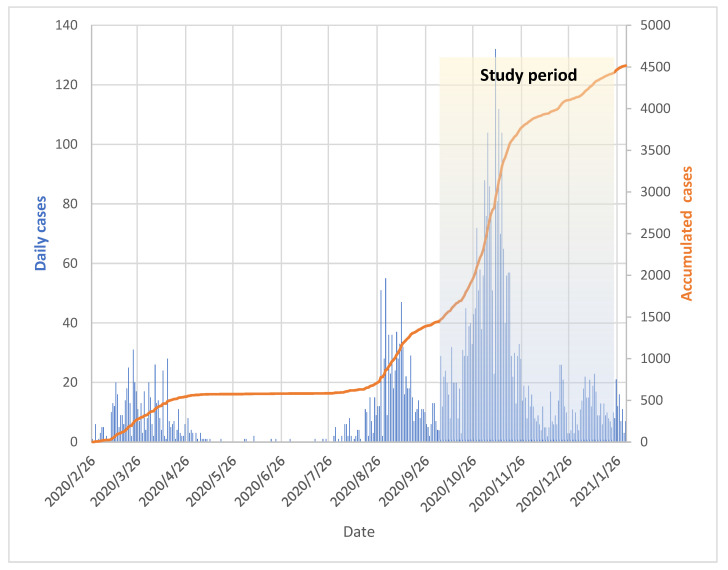
Total COVID-19 cases in the Municipality of Bergen from 26 February 2020 to 26 January 2021. The shaded area depicts the study period from 28 September 2020 to 11 January 2021. The number of daily cases is shown in blue bars. The orange line graph represents accumulated cases.

A total of 747 HCWs were included in the study, of which 394 (52, 7%) were working in hospitals, 313 (41, 9%) in nursing homes and 40 (5.4%) in the ER. The majority of the participants were female (81%). We observed a varying incidence of COVID-19 in the Municipality of Bergen during the study period (Figure 2). As shown in Table 1, there was no significant difference in non-occupational exposure to SARS-CoV-2 between HCWs working in nursing homes compared to those in hospitals/the ER (9% vs. 8%). Slightly more HCWs in hospitals/the ER (66%) had direct or indirect occupational exposure in terms of working in wards caring for COVID-19 patients, compared to nursing home HCWs (60%).

Table 2 shows the total incidence of SARS-CoV-2 positivity, and its relationship to, direct occupational exposure and type of institution (nursing home versus hospital/ER). In total, 4% (29/747) tested positive by serology. A total of 16 participants reported having tested positive for SARS-CoV-2 by RT-PCR before inclusion, of which 10 had a subsequent negative serological test. Consequently, 5% (39/747) of the participants had evidence for a prior SARS-CoV-2 infection. There was no significant difference in the rates of SARS-CoV-2 positivity in nursing homes versus hospital/ER (4% vs. 6%, *p* = 0.095), including those exposed with partial/no PPE (4% vs. 8%, *p* = 0.631).

In nursing homes, HCWs had less frequent occupational exposure to SARS-CoV-2 compared to those in hospital (16% vs. 48%, *p* < 0, 001), but, when exposed, they were less likely to use full PPE (44% vs. 81%, *p*< 0.001).

HCWs working in high-risk departments were not significantly more often infected with SARS CoV-2 than those without occupational exposure (6% vs. 3%, *p* = 0.087). Reanalyzing the subset of participants without non-occupational exposure, there was still no significant difference between those two groups (5% vs. 3%, *p* = 0.226). Stratifying for the use of PPE did not reveal significant differences in the risk of infection. Furthermore, nursing home HCWs working in high-risk settings did not contract COVID-19 significantly more often than those in hospitals/the ER did (4% vs. 8%, *p* = 0.177).

## 4. Discussion

The results from this prospective, cross-sectional study revealed that the total number of previous SARS-CoV-2 infections among nursing home HCWs was comparable to that of HCWs in hospitals/the ER, despite a significantly lower direct occupational exposure to this virus. However, it is conceivable that this similarity was at least partly a result of equal non-occupational and total occupational exposure (direct and indirect). More than 50% of the nursing home HCWs exposed to COVID-19 patients did not use full PPE, a risk factor also observed in other countries [15,16]. This finding may have several causes. Firstly, nursing home residents with COVID-19 may shed SARS-CoV-2 in their home environment in an asymptomatic or pre-symptomatic phase, or accompanied by atypical symptoms, potentially reducing the awareness of COVID-19 among HCWs [6,17,18]. Secondly, staff shortages, crowded facilities and difficulties in maintaining adequate infection control measures among patients with cognitive failure might have paved the way for a rapid and extensive transmission of SARS-CoV-2 among nursing home residents and HCWs [4,19,20]. Thirdly, the availability of PPE was lower in nursing homes than in hospitals/ERs in our region during the first phases of the pandemic, as also documented from other countries [3,4,19]. In fact, early in the pandemic, the Norwegian Ministry of Health allocated only 20% of the national stockpile of PPE to nursing homes and other primary care facilities.

A substantial variation in pooled prevalence rates of SARS-CoV-2 antibodies among HCWs has been documented in different geographical regions during the first pandemic phase; they were higher in the United States (12.4%) than in several European countries (7.7%) and East Asia (4.8%) [21]. The relatively low total seroprevalence among our HCWs probably reflects a limited transmission of SARS-CoV-2 in our community due to rigorous testing and an early lockdown, implemented on 12 March 2020.

Although nursing home HCWs were exposed to COVID-19 patients without using adequate PPE, nearly three times more often than those in hospitals/the ER, the rates of previous SARS-CoV-2 infection in this subgroup was similar. Clearly, the small numbers do not allow firm conclusions. However, we might speculate that nursing home HCWs regularly were in contact with residents who were either asymptomatic or had minimal respiratory symptoms before the diagnosis was confirmed [18]. The majority of patients in hospitals/ERs had prominent symptoms and were referred with a presumptive diagnosis of COVID-19, often during the first week of the infection, when viral load appears to be highest [22].

Although not statistically significant, we observed a higher percentage of anti-SARS-CoV-2-positive nursing home HCWs using full PPE, compared to the same group in hospitals/the ER. Again, the small numbers preclude conclusions, but it might be conceivable that nursing home personnel had received less training in the correct use of PPE than HCWs in hospitals/the ER during the first phases of the pandemic. Other potential explanations could be that only surgical face masks were available at nursing homes, which offer less protection against the transmission of SARS-CoV-2 than N95 respirators [23], or that HCWs were infected with SARS-CoV-2 through non-occupational exposure.

Among the 16 study participants who reported an RT-PCR-confirmed SARS-CoV-2 infection, 10 were negative by serology, constituting 25% of those with evidence for previous COVID-19. Data from our region during the first pandemic wave have demonstrated higher proportions of seropositivity than RT-PCR positivity among both HCWs and the general population [14,24]. Although we do not have corroborating data, participants with previous COVID-19 and negative serology might have been infected early in the first pandemic wave, more than six months prior to inclusion. Hence, as antibody titers are known to decline after infection, a prior positive PCR is indicative of true infection, despite negative serological testing. However, since PCR positivity is dependent on both the timing and technique of sampling, a prior negative PCR should not negate the interpretation of a later positive serological test, particularly as serology is more sensitive at detecting previous infection [25].

A strength of this study is that we included HCWs from both nursing homes, an ER and hospitals in a well-defined geographical region, and retrieved data on occupational exposure to COVID-19 patients/residents. Moreover, the nursing homes involved were quite similar, both regarding size, resident population, staffing and the quality of the facilities, and followed equal guidelines for infection control measures and the use of PPE.

However, our study has several limitations. The sample size was relatively small, as compared to many other SARS-CoV-2 serosurveillance studies [1,7,8], reducing the statistical power of subgroup analyses. Moreover, around 9% of the participants had non-occupational exposure to SARS-CoV-2, which might have obscured the interpretation of our data, particularly in the group of HCWs with direct exposure to COVID-19 patients. The study participants were consecutively enrolled in a period with varying incidence of COVID-19, potentially influencing the overall risk of exposure prior to serological testing. However, the level of SARS-CoV-2 transmission in our community was low-to-moderate both before and during the study period, and non-occupational exposure was reportedly similar across subgroups.

## 5. Conclusions

Our study shows that the rates of SARS-CoV-2 infection among HCWs in nursing homes and hospitals/ERs were comparable, although higher percentages of HCWs in the latter group had direct occupational exposure to SARS-CoV-2. Despite the fact that HCWs in nursing homes lacked sufficient PPE during the early phase of the pandemic, we did not find significant differences in the risk for COVID-19 of HCWs exposed to SARS-CoV-2 without full PPE between the two groups. These findings might be related to several factors, including varying patient/resident attributes, similar non-occupational exposure in the two HCW groups and a relatively small sample size.

## Figures and Tables

**Table 1:** Demographic characteristics of HCWs, by institution.

	Nursing Home (*n* = 313)	Hospital and ER (*n* = 434)	OR (95% CI)	*p*-Value
Sex (men) *	42/311 (14%)	100/433 (23%)	0.52 (0.35–0.77)	<0.001
Age (years), (median, IQR)	44 (32, 56)	41 (31, 53)	-	0.028
Non-occupational exposure **	27/298 ** (9%)	32/401 (8%)	1.15 (0.67–1.997)	0.711
Work in ward with COVID-19 patients ***	189/313 (60%)	285/434 (66%)	0.80 (0.59–1.08)	0.101

CI, confidence interval; IQR, interquartile range; OR, odds ratio. * Missing data: nursing home HCWs, *n* = 3, hospital/ER HCWs, *n* = 1. ** Close contact with individuals with suspected/confirmed COVID-19 outside institution. Missing data: nursing home HCWs, *n* = 15, hospital/ER HCWs, *n* = 33. *** Working in a nursing home, hospital ward or ER caring for COVID-19 patients, with or without direct exposure to SARS-CoV-2.

**Table 2:** Direct occupational exposure and SARS-CoV-2 positivity among HCWs, by institution.

	Nursing Home, N (%)	Hospital and ER, N (%)	OR (95% CI)	*p*-Value
Occupational exposure				
Direct exposure * total	50/310 (16)	197/407 (48)	0.20 (0.14–0.29)	<0.001
Direct exposure, full PPE **	22/50 (44)	159/197 (81)	0.19 (0.10–0.36)	<0.001
Direct exposure, partial/no PPE ***	28/50 (56)	38/197 (19)	5.33 (2.75–10.31)	<0.001
**SARS-CoV-2-positive** **during the study** period ****				
Total	11/313 (4)	28/434 (6)	0.53 (0.26–1.08)	0.095
Direct exposure, total	4/50 (8)	13/197 (7)	1.23 (0.38–3.95)	0.755
Direct exposure, full PPE	3/22 (14)	10/159 (6)	2.35 (0.59–9.31)	0.198
Direct exposure, partial/no PPE	1/28 (4)	3/38 (8)	0.43 (0.04–4.39)	0.631

CI, confidence interval; IQR, interquartile range; OR, odds ratio. * HCWs directly involved in the treatment of patients with COVID-19. Missing data for direct exposure: nursing home HCWs, *n* = 3, hospital/ER HCWs, *n* = 27. ** Full PPE: full-length gown, gloves, eye protection and facial mask. *** Partial PPE: lacking one or more components of full PPE. **** By serology and/or self-reported RT-PCR positivity prior to inclusion.

## Data Availability

The small subgroups of participants make the risk of the identification of sensitive data of individual persons possible; therefore, the data are not openly accessible.
